# Impact of osteosarcopenia in older people on prognosis following major surgery: a scoping review

**DOI:** 10.7717/peerj.20527

**Published:** 2026-01-08

**Authors:** Yshoner Antonio Silva-Diaz, Cintya Odar-Rojas, Wilson Pasten-Hidalgo, Eduardo Gallegos-Chavez, Cristian Barros-Osorio, Walter Sepúlveda-Loyola

**Affiliations:** 1 Instituto de Salud Integral Intercultural (ISI), Facultad de Ciencias de la Salud (FACISA), Universidad Nacional Toribio Rodríguez de Mendoza (UNTRM), Chachapoyas, Peru; 2 Grupo de Investigación Plantas Medicinales y Medicina Alternativa (PYMA), Centro de Investigación Plantas Medicinales, Terapias Alternativas y Comunidades Nativas y Rurales (CIPMAYCOM), Instituto de Salud Integral Intercultural (ISI), Facultad de Ciencias de la Salud (FACISA), Universidad Nacional Toribio Rodríguez de Mendoza (UNTRM), Amazon, Peru; 3 Departamento de Kinesiología, Facultad de Ciencias de la Salud, Universidad de Atacama, Copiapó, Chile; 4 Unidad de Cuidados Agudos, Hospital Dr. Sótero del Río, Programa de Especialidad de Geriatría USACH- Capredena, Santiago, Chile; 5 Grupo de Estudios del Envejecimiento (GEE), Santiago, Santiago, Chile; 6 Faculty of Health and Social Sciences, Universidad de Las Américas, Santiago, Región Metropolitana, Chile; 7 Centro de Investigación en Ciencias Biológicas y Químicas (CICBQ), Universidad de Las Américas (UDLA), Santiago, Chile

**Keywords:** Sarcopenia, Surgery, Osteopenia, Aged

## Abstract

**Purpose:**

This review aims to map the existing literature on the prevalence, diagnostic criteria, and impact of osteosarcopenia on postoperative clinical outcomes in older adults.

**Methods:**

The search for this scoping review followed the PRISMA extension guidelines across five databases (Medline, Scopus, Web of Science, Scielo, and PEDro) from their inception until August 2025. Eligible studies included older adults with osteosarcopenia who underwent major surgeries and reported clinical outcomes. Additionally, data extraction covered three themes: study and population characteristics; prevalence and impact of osteosarcopenia on post-major surgery clinical outcomes; and diagnostic criteria for osteosarcopenia.

**Results:**

A total of 164 studies were identified, of which 18 met the inclusion criteria, involving 3,235 participants aged between 60.7 and 83 years. The impact of osteosarcopenia varies depending on the type of procedure: (1) in oncological surgeries, the prevalence ranged from 12% to 44%, with reported complications including prolonged surgical time and reduced survival; (2) in orthopedic surgeries, prevalence ranged from 28% to 100%, with issues such as delayed recovery and increased mortality; and (3) in cardiovascular and gastrointestinal surgeries, the prevalence of osteosarcopenia ranged from 6.5% to 38.5%, associated with delayed wound healing and higher infection rates. Diagnostic approaches to osteosarcopenia showed substantial heterogeneity, most frequently relying on skeletal muscle mass index and bone mineral density, but applying different cutoff values.

**Conclusions:**

The prevalence of osteosarcopenia differs across populations and surgical contexts. This syndrome represents an important risk factor for adverse postoperative outcomes in older adults undergoing major surgery. Furthermore, considerable variability persists in the diagnostic criteria employed, underscoring the need for standardized definitions to improve clinical applicability and comparability across studies.

## Introduction

Osteosarcopenia is defined as a geriatric syndrome characterized by the coexistence of osteoporosis and sarcopenia (Sepúlveda-Loyola et al., 2020), its global prevalence among older adults is 21% (Huang et al., 2023), emerging as a significant public health concern (Drey et al., 2015; Mella De Cuevas et al., 2022). Osteosarcopenia may exacerbate the vulnerability of older adults by further compromising musculoskeletal integrity and physiological reserves (Hassan & Duque, 2017; Kirk, Zanker & Duque, 2020). This geriatric syndrome not only heightens the risk of fractures and functional decline, but also carries significant clinical implications for prognosis during hospitalization, often due to exacerbations of chronic diseases, fractures, or surgical interventions (Fagundes Belchior et al., 2020; Kolbaşı& Demirdağ, 2020; Pourhassan et al., 2024).

As the global population continues to age, the number of older adults undergoing major surgeries including cardiac procedures, fracture repair, and oncological surgeries has increased substantially (Aucoin & McIsaac, 2019). Surgical outcomes in this population are influenced not only by chronological age but also by geriatric syndromes that exacerbate vulnerability to stressors. Among these, frailty and sarcopenia have been consistently associated with adverse postoperative outcomes, including higher rates of mortality, complications, and prolonged hospital stays (Checa-Lopez et al., 2023; Liu et al., 2024). Moreover, patients with osteosarcopenia appear to be at particularly high risk, often experiencing extended recovery periods, greater dependency, and elevated rates of morbidity and mortality (Abe et al., 2024; Wang et al., 2024). The prevalence of osteosarcopenia among older adults undergoing surgery has been reported to range between 15% and 42% (Hirase et al., 2024; Solla-Suarez et al., 2024). Importantly, preoperative features of this syndrome, such as reduced bone mineral density, diminished muscle mass, and impaired muscle strength, may elevate the likelihood of intraoperative and postoperative complications, including delayed wound healing and prolonged hospitalization (Veronese et al., 2024).

The connection between osteosarcopenia and surgical recovery is complex and involves multiple factors. This syndrome may influence preoperative risk assessments, postoperative complications, and the outcomes of long-term rehabilitation (Bae & Moon, 2020; Veronese et al., 2024). Recent studies have demonstrated that older adults with osteosarcopenia face up to a tenfold increased risk of being classified as frail (Chew et al., 2020; Saeki et al., 2020). This strong association suggests that osteosarcopenia may contribute to the clinical expression of frailty, since both conditions share common pathophysiological mechanisms such as chronic inflammation, hormonal dysregulation, and musculoskeletal decline (Mella De Cuevas et al., 2022; Shafiee et al., 2024). Importantly, frailty itself has been consistently identified as a predictor of adverse surgical outcomes, including increased complications, longer hospital stays, and higher mortality (Solla-Suarez et al., 2024). Therefore, although osteosarcopenia is widely recognized for affecting surgical outcomes, to the best of our knowledge, there is a lack of information in the literature regarding how osteosarcopenia impacts different types of surgery in older patients. This leads to the following question: What is the prevalence, impact, and diagnostic criteria of osteosarcopenia in postoperative patients who have undergone various major surgeries? Therefore, identifying this question, along with the gaps in current knowledge, would highlight areas for future research and support the optimization of surgical care in the aging population. Consequently, this review aims to map the existing literature on the prevalence, diagnostic criteria, and impact of osteosarcopenia on postoperative clinical outcomes in older adults.

## Materials & Methods

This scoping review was reported and summarized using the Preferred Reporting Items for Systematic Reviews and Meta-Analyses (PRISMA) recommendations extension for scoping reviews (Munn et al., 2018; Tricco et al., 2018). The protocol for this exploratory review was registered in the Open Science Framework (OSF) under the code OSF.IO/SNRPU (Sepúlveda-Loyola, 2024), (available at: https://osf.io/snrpu/).

The scoping review search was conducted across five databases Medline, Scopus, Web of Science, Scielo and PEDro from inception until August 2025. The search strategy, including all identified keywords, Medical Subject Headings (MeSH) and Descriptores en Ciencias de la Salud (DeCS) terms in English, Portuguese and Spanish (Osteosarcopenia; Osteosarcopenic, General Surgery, Thoracic Surgery, Acute Care Surgery, Hospitalization, Orthopedic Procedures, Minor Surgical Procedures and Enhanced Recovery After Surgery) according to the database requirement for search strategy (Table S1). Additionally, a manual search was conducted in the reference lists of each included article to identify potential additional studies.

### Search strategy and inclusion criteria

We used the patient, concept, context (PCC) framework (Pollock et al., 2023). Studies that met the following criteria were considered: (1) Population: Older adults diagnosed with osteosarcopenia, defined as the coexistence of sarcopenia and osteopenia/osteoporosis, according to criteria described in the methodology section; (2) Design: Experimental studies, observational studies, analytical studies, and qualitative studies; (3) Concept: Clinical outcomes assessed before and after major surgery, defined as complex surgical interventions involving internal organs and requiring general or regional anesthesia defined as complex surgical interventions involving internal organs and requiring general or regional anesthesia and involving the opening of major body cavities (Martin et al., 2020); (4) Context: From all geographic locations and regions in the world. (5) Language: Studies in all languages with full article access were included, so that team members proficient in Spanish and Portuguese could translate them into English. Additionally, the following studies were excluded: (1) Design: Theses, reports, reviews and case conferences; (2) Diagnostic omission: Studies that did not describe the diagnosis of osteosarcopenia.

The information in this scoping review was organized in the following topics:

1- Characteristics of the studies and the included population.
2- Prevalence and impact of osteosarcopenia on clinical outcomes after and before different major surgeries.
3- Criteria used to diagnose osteosarcopenia in all studies

### Data extraction

Two independent reviewers (CO and YS) assessed the eligibility of articles based on title and abstract, categorizing citations as ‘include,’ ‘exclude,’ or ‘maybe.’ Those deemed as ’include’ or ’maybe’ were further reviewed in full text. Any disagreements were resolved through consultation with a third independent advisor (WSL). The total number of studies was stored in a single file. Results from the database searches were cross-checked using Mendeley, and duplicates were excluded.

Data extraction was performed independently by two team members (EGC and CBO) using standardized templates adapted to meet the objectives of the study. Any disagreement regarding data extraction or eligibility assessment was resolved through consultation with a third independent advisor (WSL). We used a standardized extraction sheet to collect the following information from each included study: first author and year of publication, study design, country of origin, study objective, sample size, sex distribution, mean or median age, diagnostic criteria for osteosarcopenia, prevalence of osteosarcopenia, comorbidities, type of surgery, recruitment approach, postoperative complications, and outcome measures.

### Data analysis

The analysis was descriptive in nature, consistent with the objectives of a scoping review. Extracted data were summarized using frequency counts, descriptive statistics, and narrative synthesis. To enhance the mapping of evidence, data were presented in summary tables and visualized through figures illustrating: (1) sex distribution of the included populations, (2) geographic distribution of the studies, (3) diagnostic criteria used for osteosarcopenia, (4) prevalence of osteosarcopenia across surgical contexts, and (5) postoperative complications stratified by surgery type. The figures were created using GraphPad Prism 10.4.2 (GraphPad, 2025); Flourish (Make a Sankey Diagram without Coding — Flourish, 2025); and ArcGIS Pro 3.5 (ArcGIS Pro-ArcGIS Pro, 2025).

## Results

### Characteristics of the studies and the included population

**Figure 1:**
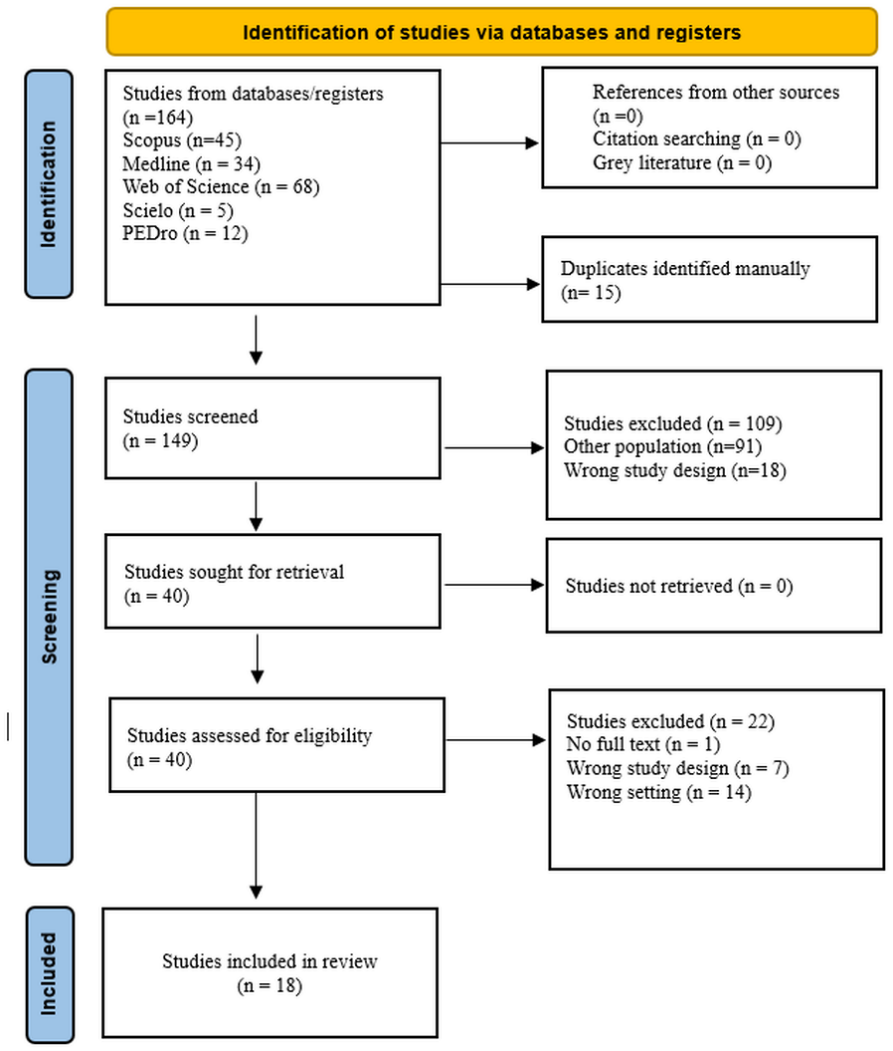
PRISMA flow diagram of article selection.

**Figure 2:**
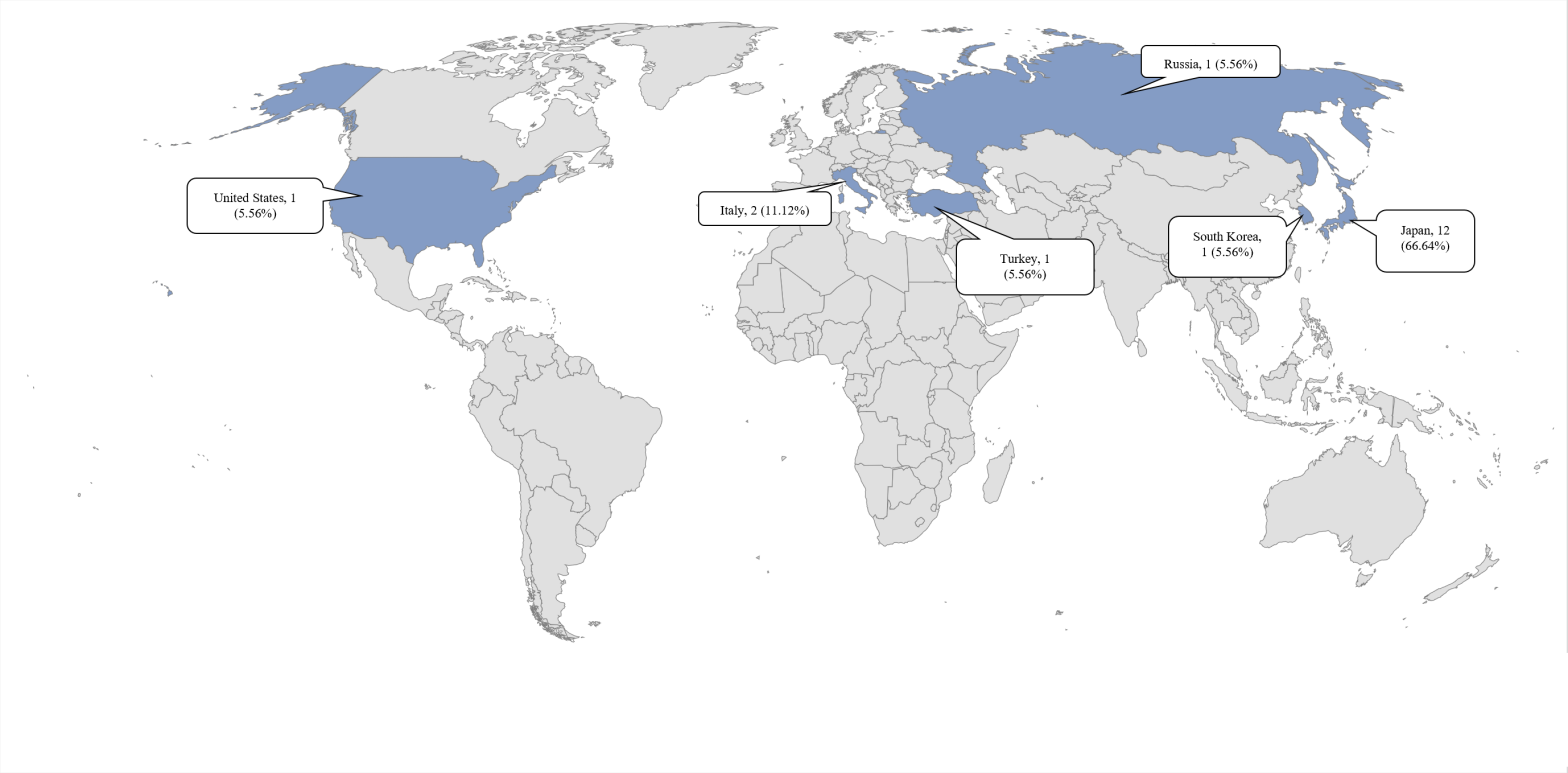
Geographic distribution of the included studies. Note: The map shows the geographic distribution, highlighting the countries where the studies were conducted (in blue). The labels indicate the proportion relative to the total number of selected studies.

Eighteen studies were identified that met the established inclusion and exclusion criteria (Fig. 1). Among the analyzed articles, 14 retrospective studies were identified (77.7%) (Abe et al., 2024; Abe et al., 2023; Bae & Moon, 2020; Fujimoto et al., 2024; Fukushima et al., 2024; Furukawa et al., 2021; Furukawa et al., 2023; Kara & Ozturk, 2023; Matsumoto et al., 2024; Takano et al., 2023; Taniai et al., 2023; Yanagaki et al., 2023; Yanagaki et al., 2024), two prospective studies (11.1%) (Bottai et al., 2023; Haffer et al., 2023), one cohort study (5.5%) (Bazdyrev et al., 2022) and 1 cross-sectional study (5.5%) (Di Monaco et al., 2022). Geographically, the majority of the studies were conducted in Asia. Among these, 12 studies (66.6%) were primarily carried out in Japan (Abe et al., 2024; Abe et al., 2023; Fujimoto et al., 2024; Fukushima et al., 2024; Furukawa et al., 2021; Furukawa et al., 2023; Hirase et al., 2025; Matsumoto et al., 2024; Takano et al., 2023; Taniai et al., 2023; Yanagaki et al., 2023; Yanagaki et al., 2024), one study (5.5%) in South Korea (Bae & Moon, 2020), and one study (5.5%) in Turkey (Kara & Ozturk, 2023). In Europe, two studies (11.1%) were conducted in Italy (Bottai et al., 2023; Di Monaco et al., 2022) and 1 study (5.5%) in Russia (Bazdyrev et al., 2022). Additionally, there was one study (5.5%) conducted in North America, specifically in the United States (Haffer et al., 2023) (Fig. 2).

**Figure 3:**
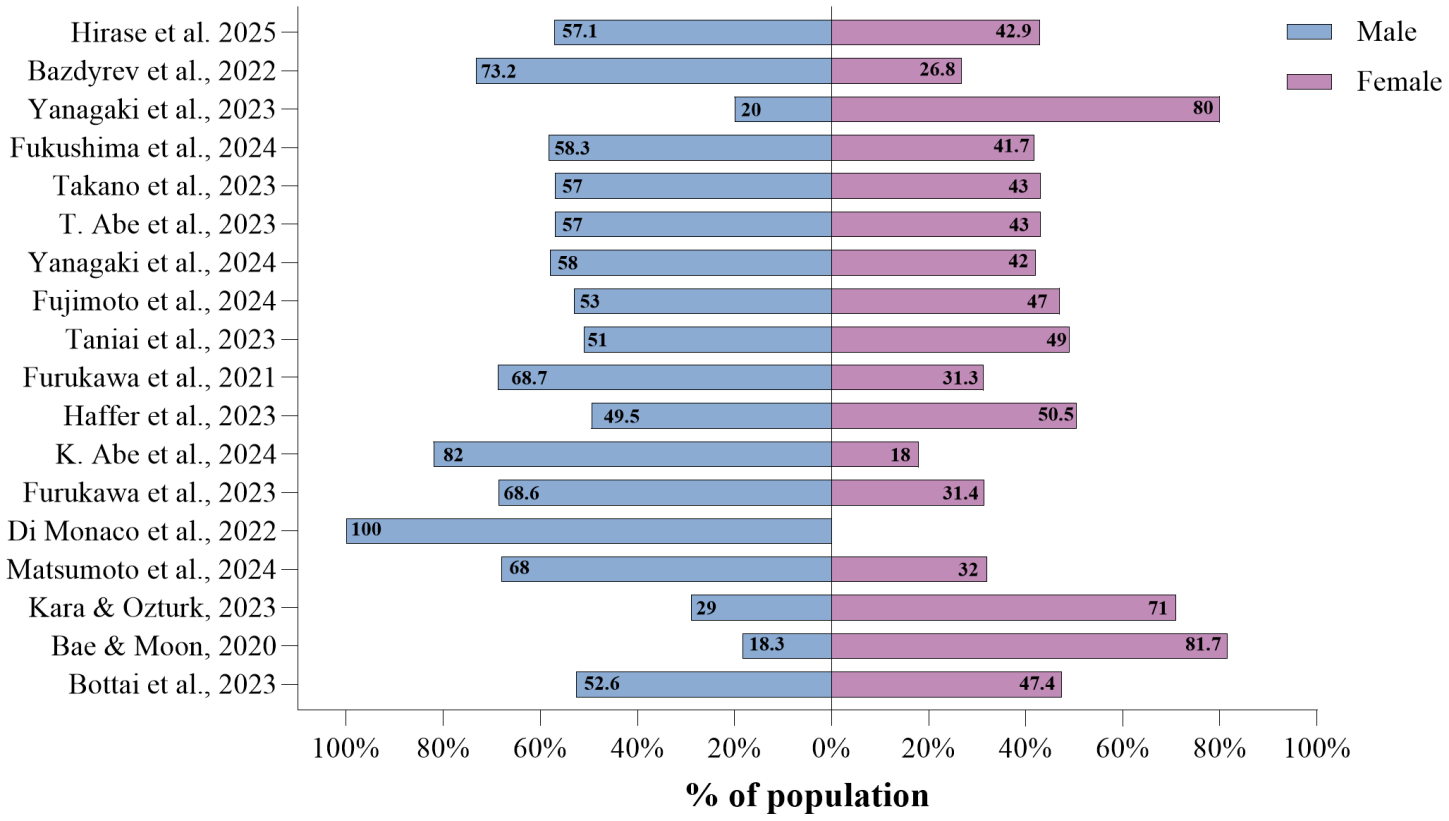
Percentage of male and female participants by study. Note: In this horizontal bar chart, the values represent the percentage of male (blue) and female (purple) participants reported in the selected studies (Hirase et al., 2025; Bazdyrev et al., 2022; Yanagaki et al., 2023; Fukushima et al., 2024; Takano et al., 2023; Abe et al., 2023; Yanagaki et al., 2024; Fujimoto et al., 2024; Taniai et al., 2023; Furukawa et al., 2021; Haffer et al., 2023; Abe et al., 2024; Furukawa et al., 2023; Di Monaco et al., 2022; Matsumoto et al., 2024; Kara & Ozturk, 2023; Bae & Moon, 2020; Bottai et al., 2023).

The total sample consisted of 3,235 participants across the 18 studies, of which 14 studies reported higher percentages of male participants, ranging from 18.3% to 100% (Fig. 3) (Bae & Moon, 2020; Di Monaco et al., 2022). The sample size varied considerably between the studies, ranging from 19 to 594 participants (Bottai et al., 2023; Fujimoto et al., 2024). The mean age range varied between 60.7 and 83 years (Bae & Moon, 2020; Haffer et al., 2023). The prevalence of osteosarcopenia showed considerable variability, ranging from 6.5% to 100% (Bazdyrev et al., 2022; Bottai et al., 2023), reflecting the differences in population characteristics and the methodologies employed. There proportion of women with osteosarcopenia ranged from 31.4% to 81.7% (Bae & Moon, 2020; Furukawa et al., 2023) (Table 1).

### Prevalence and impact of osteosarcopenia on clinical outcomes before and after different major surgeries

**Figure 4:**
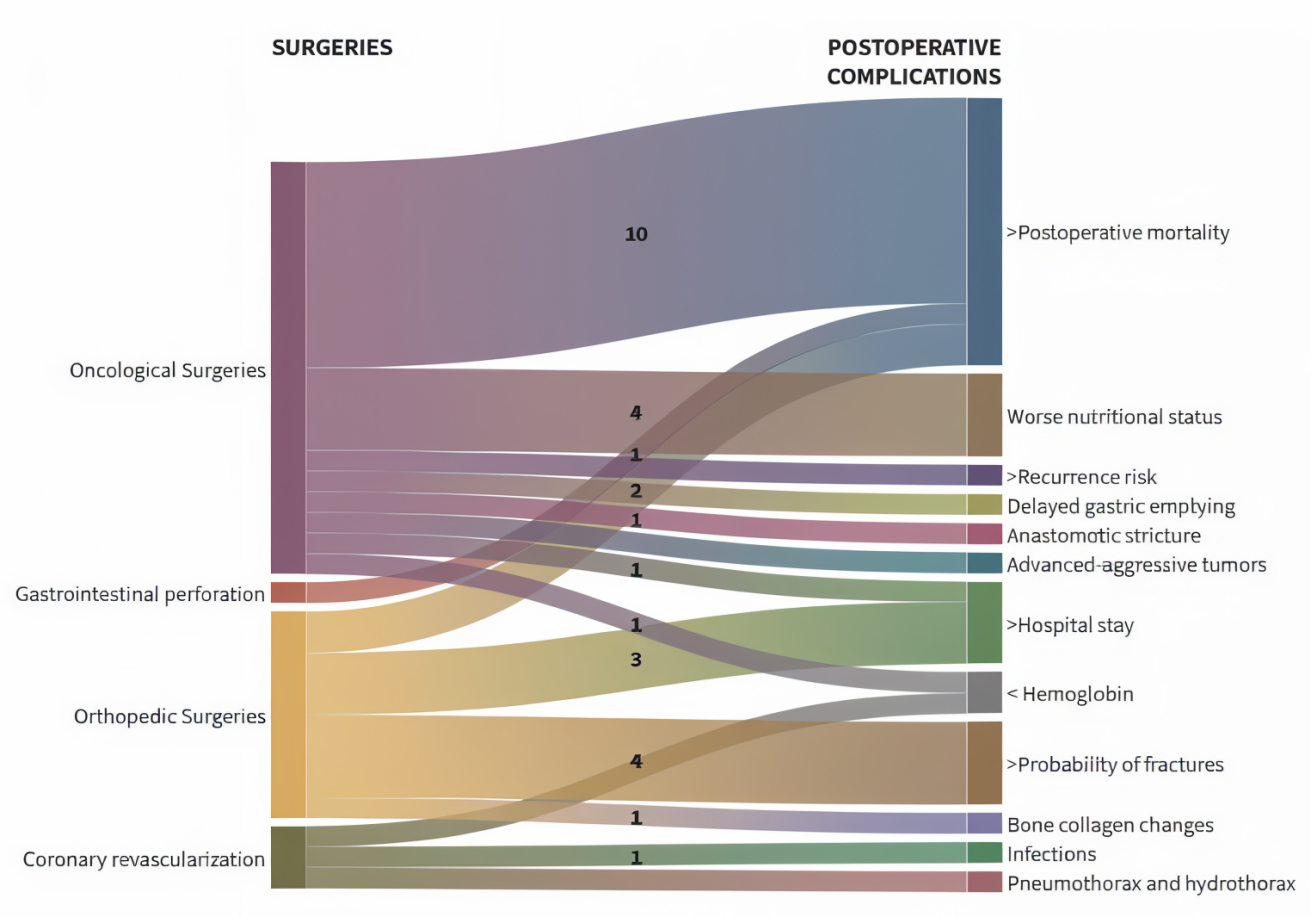
Postoperative complications reported by type of surgery. Note: The Sankey diagram links types of surgeries to postoperative complications. The numbers on each band represent the number of studies reporting these complications.

**Figure 5:**
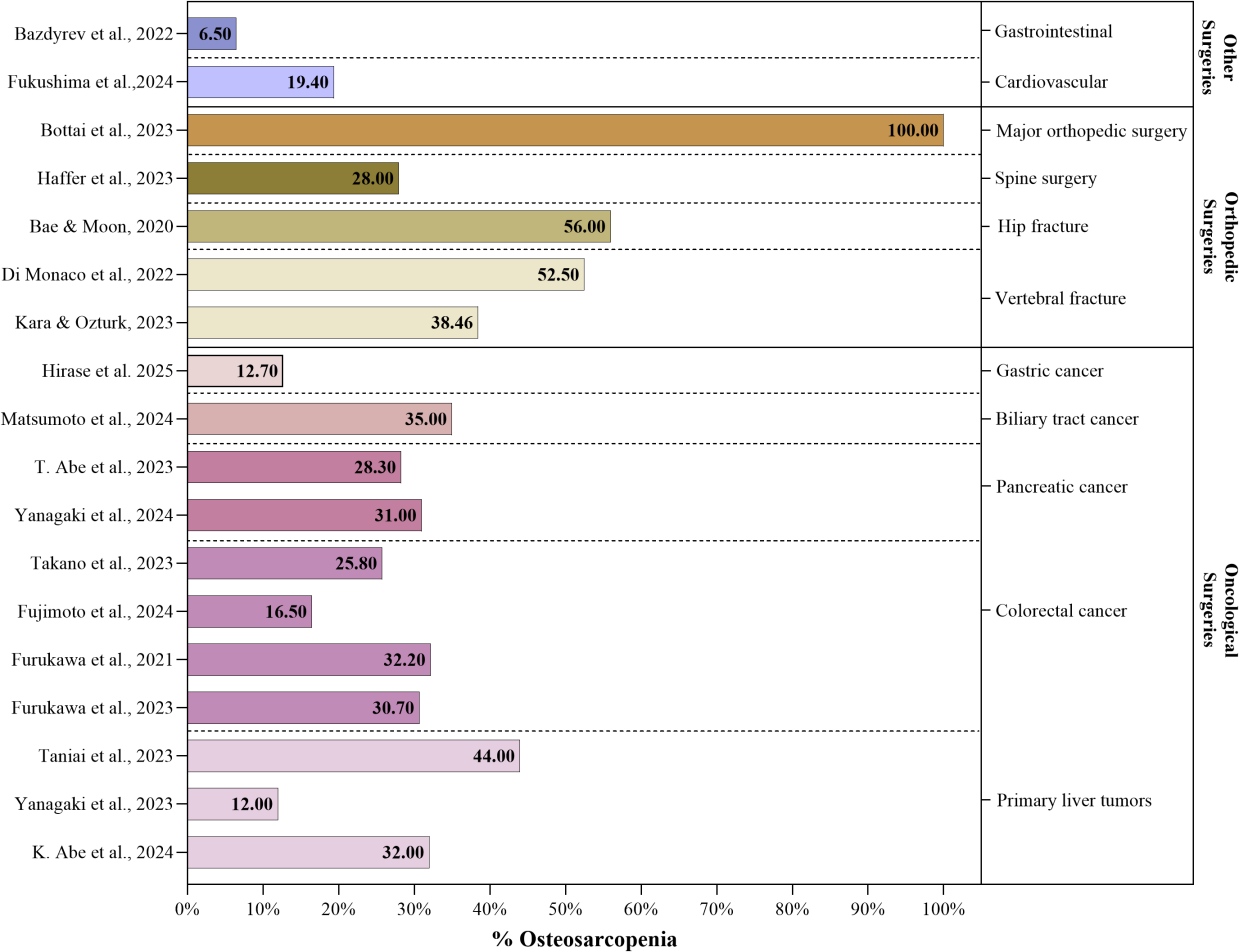
Percentage distribution of osteosarcopenia by type of surgery. Note: The figure shows the prevalence of osteosarcopenia in oncologic, orthopedic, gastrointestinal, and cardiovascular surgeries. The numerical values on the bars represent the percentages reported in each study (Hirase et al., 2025; Bazdyrev et al., 2022; Yanagaki et al., 2023; Fukushima et al., 2024; Takano et al., 2023; Abe et al., 2023; Yanagaki et al., 2024; Fujimoto et al., 2024; Taniai et al., 2023; Furukawa et al., 2021; Haffer et al., 2023; Abe et al., 2024; Furukawa et al., 2023; Di Monaco et al., 2022; Matsumoto et al., 2024; Kara & Ozturk, 2023; Bae & Moon, 2020; Bottai et al., 2023).

Eleven studies were identified, involving a total of 2,196 participants oncological patients, in which the prevalence of osteosarcopenia ranged from 12% (Yanagaki et al., 2023) to 44% (Taniai et al., 2023). Additionally, among the main complications associated with these surgeries, postoperative mortality was highlighted by eight authors in the reviewed literature (Abe et al., 2024; Abe et al., 2023; Fujimoto et al., 2024; Furukawa et al., 2021; Matsumoto et al., 2024; Takano et al., 2023; Taniai et al., 2023; Yanagaki et al., 2023), followed by four authors who reported a deterioration in nutritional status (Fujimoto et al., 2024; Matsumoto et al., 2024; Taniai et al., 2023; Yanagaki et al., 2024) (Fig. 4). In primary liver tumors, the prevalence of osteosarcopenia ranged from 12% to 44% (Taniai et al., 2023; Yanagaki et al., 2023), showing that disease-free survival in patients with osteosarcopenia was 13.4 months, compared to 21.2 months in those without osteosarcopenia (Yanagaki et al., 2023). In colorectal cancer, which included studies with 118, 140, 594, and 325 patients (Fujimoto et al., 2024; Furukawa et al., 2021; Furukawa et al., 2023; Takano et al., 2023), the prevalence of osteosarcopenia ranged from 16.5% to 32.2% (Fujimoto et al., 2024; Takano et al., 2023). It was also associated with a higher rate of complications of grade ≥3 according to the Clavien-Dindo classification and worse postoperative survival outcomes (Fujimoto et al., 2024; Takano et al., 2023). In patients with pancreatic cancer who underwent procedures such as pancreaticoduodenectomy and distal pancreatectomy, the sample consisted of 183 to 265 patients (Abe et al., 2024; Yanagaki et al., 2023), with osteosarcopenia prevalence ranging from 28.3% to 31% (Abe et al., 2024; Yanagaki et al., 2023); these patients exhibited a higher in-hospital mortality rate and prolonged surgical times (median of 490 min) (Yanagaki et al., 2023). In patients with biliary tract cancer, involving 138 patients, the prevalence of osteosarcopenia was 35%, which was associated with a higher risk of postoperative complications, more aggressive tumors, and poorer nutritional status (Matsumoto et al., 2024). Finally, one study was conducted in 63 patients with gastric cancer, reporting a higher incidence of anastomotic stenosis and a high prevalence of mortality in patients with osteosarcopenia (Hirase et al., 2024) (Fig. 5).

**Table 1:** Characteristics of the study included in the scoping review.

**Author and year**	**Study desing**	**Country of the study**	**Objetive of the study**	**Sample size total** **(n)**	**Age (years)**	**Female (n and %)**	**Osteosarcopenia criteria**	**Osteosarcopenia prevalence (n y %)**	**Other comorbidities of the population**	**Surgery intervention**	**Postoperative complications**
Bottai et al. (2023)	Prospective	Italy	The goal is to analyze the prevalence of osteosarcopenia related to inactivity and its impact on health.	19	15–85	9 (47.4%)	-**Bone Metabolism:** Blood tests upon admission (calcium, phosphorus, vitamin D, *etc*.) -**Muscle Biopsies:** Biceps in upper limbs, quadriceps in lower limbs. -**Histological Analysis:** Staining and specialized evaluation (Hematoxylin-Eosin at 20x).	19 (100%)	-Severe hipovitaminosis -Hypocalcemia	-Resection or custom-made prosthesis. -Resection and reconstruction with transplant. -Conducted for oncological reasons.	-** Preoperative Inactivity:** Average of 9.05 months.
Bae & Moon (2020)	Retrospective	South Korea	Evaluate how muscle mass and bone density influence postoperative outcomes and fracture rates in elderly patients.	126	83	103 (81.7%)	-**Sarcopenia:** Using PMI. Total area of the psoas muscles divided by height squared -**Osteopenia/ osteoporosis:** Through CT scans; a T-score less than −2.5 indicates osteosarcopenia.	58 (46%)	-Hip fracture	-Internal fixation -Bipolar arthroplasty or total hip arthroplasty	-**Functional Outcomes:** Lower scores on the Barthel Index and HHS. -**Fractures:** Higher incidence of fractures.
Kara & Ozturk (2023)	Retrospective	Turkey	This study explored how osteosarcopenia affects second fractures and mortality in older patients.	104	60–92	74 (71%)	-**Sarcopenia:** Using PMI evaluated at the level of the third lumbar vertebra. -**Osteopenia/ osteoporosis:** Through Hounsfield Unit Values measured at third lumbar vertebra.	40 (38.46%)	- Thoracic or lumbar vertebral fracture	-Bilateral transpedicular injection of polymethylmethacrylate. -Biopsy for pathological evaluation.	-**Mortality:** Higher mortality in osteosarcopenia -**Fractures:** Higher likelihood of fractures (mean time between fractures 15.62 ± 16.18 months)
Matsumoto et al. (2024)	Retrospective	Japan	Examine the impact of preoperative osteosarcopenia on recurrence and prognosis in extrahepatic bile duct cancer.	138	71	44 (32%)	-**Sarcopenia:** using PMA measured at the third lumbar vertebra using CT. -**Osteopenia:** Low BMD in 11th thoracic vertebra based on age, through CT scans.	48 (35%)	-Vascular disease Diabetes -Tumor location: PHCC; DCC; GBC y AC	HBS PD	-**Mortality:** survival range is 20.6% in those individuals with osteosarcopenia and 57.4% without it. -**Other Complications:** Predictor of poorer outcomes in bile duct cancer. More aggressive tumors and poor nutritional status in those with osteosarcopenia.
Di Monaco et al. (2022)	Cross-sectional	Italy	Assess whether definitions of low appendicular lean mass (aLM) include men with low bone mineral density (BMD) and hip fractures.	80	81.4	0	-**Sarcopenia:** Low aLM according to FNIH criteria. -**Osteopenia/ osteoporosis:** Low BMD in the femoral neck and total hip according to WHO criteria	42 (52.5%)	-Correction of aLM -Vertebral fracture	-Post-fracture	-**Mortality:** Higher mortality in individuals with osteosarcopenia -**Recovery time:** poor recovery after hip fracture surgery -**Other Complications:** Increase in falls and fractures,
Furukawa et al. (2023)	Retrospective	Japan	The study evaluates the impact of occult vertebral fractures in patients with liver metastases after hepatectomy.	140	66	44 (31.4%)	-**Sarcopenia:** low PMA -**Osteopenia:** Low BMD.	43 (30.71%)	-Symptomatic vertebral fracture. -Colorectal liver metastases	-Hepatic resection	-** Mortality:** The survival of patients with osteosarcopenia is lower compared to those without the condition.
Abe et al. (2024)	Retrospective	Japan	Identify factors that affect the prognosis of patients with Barcelona Cancer Liver Classification stage A hepatocellular carcinoma	102	74	6 (18%)	-**Sarcopenia:** low SMI (38 cm^2^/m^2^ for women and 42 cm^2^/m^2^ for men). -**Osteopenia/ osteoporosis:** BMD using the cut off points 160 HU.	33 (32%)	-HBV -HCV -BCLC	Hepatic resection RFA	-**Mortality:** Lower survival and higher risk of recurrence (2.44 times) and mortality (3.23 times) in patients with stage A hepatocellular carcinoma -**Recovery time**: longer hospital stays
Haffer et al. (2023)	Prospective cross-sectional	United States	Investigate whether the accumulation of AGEs negatively affects paraspinal muscle composition	107	60.7	54 (50.5%)	-**Bone biopsies:** To analyze the accumulation of advanced glycation end products, which indicate reduced bone quality. -**Sarcopenia:** Using MRI in paraspinal muscles, such as the psoas, multifidus, and erector spinae muscles, -**Osteopenia/ osteoporosis**: BMI using CT,	30 (28%)	-Fracture	-Spinal surgery for fracture management	-**Other Complications:**: Bone collagen modifications (fAGEs). AGEs increase fragility.
Furukawa et al. (2021)	Retrospective	Japan	The study examines how preoperative osteosarcopenia influences outcomes in CRLM patients after liver resection.	118	67.5	37 (31.3%)	-**Sarcopenia:** Low PMA at the third lumbar vertebra. -**Osteopenia:** Low BMD at the 11th thoracic vertebra.	38 (32.2%)	- HCC: etapa A (BCLC A)	-Hepatectomy -Percutaneous transhepatic portal embolization.	-**Mortality:** Overall survival is worse in patients with osteosarcopenia, especially in men, and compared to those with only osteopenia or sarcopenia.
Taniai et al. (2023)	Retrospective cohort study	Japan	This study examines how osteosarcopenia affects prognosis in patients with intrahepatic cholangiocarcinoma.	41	63	20 (49%)	-**Sarcopenia:** Measured by PMA at third lumbar vertebra using CT scans -**Osteopenia:** Measured by BMD at 11th thoracic vertebra using CT scans.	18 (44%)	-HBs-Ag -HCV-Ab	- IHCC	-**Mortality:** Osteosarcopenia reduces survival in IHCC -**Other complications:** Reduced BMI, BMD, and PMA. Along with high CEA and advanced T stage
Fujimoto et al. (2024)	Retrospective cohort study	Japan	To assess if osteosarcopenia affects CRC outcomes after surgery.	594	68	279 (47%)	-**Sarcopenia:** SMI at third lumbar vertebra using CT (<30 cm^2^/m^2^ for women, <40 cm^2^/m^2^ for men) -**Osteopenia:** BMD at Th11 11th thoracic vertebra using CT scans.	98 (16.5%)	-Tumor stage I,II and III.	-Laparoscopic Surgery	-**Mortality:** lower 5-year survival in patients with osteosarcopenia. -**Other complications:** Reduced BMI, hemoglobin, and PNI.
Yanagaki et al. (2024)	Retrospective cohort study	Japan	To assess the prognostic impact of osteosarcopenia in PDAC patients undergoing pancreatic resection.	183	71	72 (42%)	-**Sarcopenia:** SMI at third lumbar vertebra using CT -**Osteopenia:** BMD at Th11 11th thoracic vertebra using CT scans.	61 (31%)	Tumor -Preoperative -Biliary Drainage	-TP -DP -PD	-**Other complications:** Increases postoperative complications. Osteosarcopenia reduces DFS and OS. -**Nutritional Status:** Associated with lower PLR, indicating poor nutrition and inflammation.
Abe et al. (2024)	Retrospective cohort study	Japan	Assess the impact of preoperative osteosarcopenia on survival prediction in PDAC patients undergoing curative surgery.	265	69	114 (43%)	-**Sarcopenia:** SMI at third lumbar vertebra using CT -**Osteopenia:** BMD at Th11 11th thoracic vertebra using CT scans; age-adjusted cutoff values.	75 (28.3%)	Preoperative diabetes -Tumor Stage: 91% resectable, 9% borderline resectable (per UICC 8th edition).	-PD -DP	-**Mortality:** the overall survival was 23 months with osteosarcopenia compared to 48 months without it (HR: 1.98). -**Disease-Free Survival:** 13.4 months with osteosarcopenia vs. 21.2 months without (HR: 1.53). -**Complications:** Higher risk off all postoperative complications
Taniai et al. (2023)	Retrospective	Japan	This study explored the prognostic impact of osteosarcopenia in older adults undergoing curative resection for colorectal cancer.	325	76	140 (43%)	-**Sarcopenia:** SMI at third lumbar vertebra using CT -**Osteopenia:** BMD below age standards.	73 (25.8%)	Colorectal cancer -Preoperative OSP	-Surgical resections to treat colorectal cancer	-** Mortality:** Worse survival outcomes
Fukushima et al. (2024)	Retrospective cohort study	Japan	Evaluate the impact of preoperative osteosarcopenia on outcomes after emergency gastrointestinal surgery.	216	73	90 (41.7%)	-**Sarcopenia:** using PMA measured at the third lumbar vertebra using CT. -**Osteopenia:** BMD at Th11 11th thoracic vertebra using CT scans.	42 (19.4%)	-Conditions: Cardiovascular, respiratory, renal, diabetes, and cerebrovascular. -GI Perforation: Stomach/duodenum, small intestine, colon/rectum.	- Emergency surgical interventions	-**Mortality:** Higher in mortality during hospitalization on patients with osteosarcopenia. -**Severe Complications:** Increased with osteosarcopenia. -**Prognostic Factor:** Osteosarcopenia predicts worse outcomes better than sarcopenia or osteopenia alone.
Yanagaki et al. (2023)	Retrospective cohort study	Japan	Assess the impact of preoperative osteosarcopenia on prognosis in HCC patients undergoing liver resection.	227	69	181 (80%)	-**Sarcopenia:** SMI at third lumbar vertebra using CT -**Osteopenia:** BMD at Th11 11th thoracic vertebra using CT scans.	27 (12%)	-HBs-Ag -HCV-Ab	-Type of resection -Partial resections	-**Complications:** Osteosarcopenia reduces DFS and OS. -**Independent Factor:** Osteosarcopenia predicts lower survival.
Bazdyrev et al. (2022)	Cohort-study	Russia	Analyze complications in coronary artery disease patients with musculoskeletal disorders undergoing CABG.	387	66	104 (26.8%)	-**Sarcopenia:** Diagnosed by EWGSOP 2019 criteria. -**Osteopenia:** BMD according to WHO 2008 criteria for older adults.	25 (6.5%)	-Hypertension -Angina pectoris -Diabetes mellitus Stroke,	-Coronary artery bypass grafting (CABG)	-**Revascularization:** Higher in sarcopenia and osteosarcopenia. -**Infections:** More frequent in osteosarcopenia. -**Other Complications:** Higher in sarcopenia and osteosarcopenia (hemorrhage, pneumothorax, hydrothorax).
Hirase et al. (2025)	Retrospective observational study	Japan	To evaluate the effects of SNNS and conventional gastrectomy on changes in BMD and skeletal muscle mass in patients with EGC.	63	67	27 (42,9%)	-**Sarcopenia:** SMI calculated from CT images of the abdomen. -**Osteopenia:** It is assessed using BMD in the iliac bone at the level of the T11 vertebra.	8 (12.7%)	-Tumor stages 0-I	Laparoscopic Local Resection Laparoscopic Distal Gastrectomy	-**Mortality:** in the laparoscopic local resection group, the risk of death over-5 years was 5,6% while in the laparoscopic distal gastrectomy group the risk of death was 0%. -**Complications:** Delayed gastric emptying and anastomotic stricture. **Osteosarcopenia prevalence:** Preoperative: 8/63 (12.7%) → 4/21 LLR, 4/42 LDG5 years postoperative: 24/63 (38.1%) → 6/21 LLR (28.6%), 18/42 LDG (42.9%) (*P* = 0.25)

**Notes.**

AC Ampullary carcinoma aLM Appendicular Lean Mass BCLC Barcelona Clinic Liver Cancer BCLC A Early Stage of Cancer BMD Bone Mineral Density CABG Coronary Artery Bypass Grafting CEA Carcinoembryonic Antigen CT Computed Tomography DCC Distal Cholangiocarcinoma DFS Disease-free survival DP Distal Pancreatectomy EGC Early Gastric Cancer EWGSOP European Working Group on Sarcopenia in Older People fAGES Fluorescent Advanced Glycation Endproducts GBC Gallbladder Carcinoma GI Gastrointestinal Perforation HBS Major Hepatobiliary Resection HBs-Ag Hepatitis B Surface Antigen HCC Hepatocellular Carcinoma HCV Hepatitis C Virus HCV-Ab ] Hepatitis C Virus Antibodies HHS Harris Hip Score HR Hazard Ratio HU Hounsfield Units IHCC Intrahepatic Cholangiocarcinoma LDG Laparoscopic Distal Gastrectomy LLR Laparoscopic Local Resection MRI Magnetic Resonance Imaging OS Overall Survival PD Pancreaticoduodenectomy PDAC Pancreatic Ductal Adenocarcinoma PHCC Perihilar Cholangiocarcinoma PLR Platelet-to-Lymphocyte Ratio PMA Psoas Muscle Mass Area PMI Psoas Mass Index PNI Prognostic Nutritional Index RFA Radiofrequency Ablation SMI Skeletal Muscle Index SNNS Sentinel Node Navigation Surgery TP Total Pancreatectomy UICC Union for International Cancer Control WH Foundation for the National Institutes of Health

In patients undergoing surgery for fractures, five studies were found, including a total of 576 patients, among whom the prevalence of osteosarcopenia ranged 28% (Haffer et al., 2023) to 100% (Bottai et al., 2023). Similarly, the highest likelihood of fractures was mentioned as a relevant complication in four of the reviewed studies (Bae & Moon, 2020; Di Monaco et al., 2022; Haffer et al., 2023; Kara & Ozturk, 2023) (Fig. 4). Among elderly patients with hip fractures, the percentage of osteosarcopenia ranges from 46% to 52.5% (Bae & Moon, 2020; Di Monaco et al., 2022), showing a higher incidence of postoperative complications, an increased risk of new fractures and physical disability, slower postoperative recovery, a greater risk of falls, and elevated mortality rates (Bae & Moon, 2020; Di Monaco et al., 2022). In the case of vertebral fractures, the prevalence of osteosarcopenia in these patients ranges from 28% to 38.5% (Haffer et al., 2023; Kara & Ozturk, 2023), resulting in rates of severe postoperative complications, such as higher in-hospital mortality, fat infiltration in paraspinal muscles, increased bone fragility, and poorer overall health outcomes (Haffer et al., 2023; Kara & Ozturk, 2023). Likewise, osteosarcopenic patients who underwent major orthopedic surgeries showed that the main finding was unilateral osteoporosis associated with district osteosarcopenia in 2 out of 3 bone density scans (Bottai et al., 2023) (Fig. 5).

In cardiovascular and gastrointestinal surgeries, one study was found for each type of surgery, with a total of 216 (Fukushima et al., 2024) and 387 participants (Bazdyrev et al., 2022), in which the prevalence of osteosarcopenia ranged from 6.5% (Bazdyrev et al., 2022) to 19.4% (Fukushima et al., 2024) respectively (Fig. 5). In patients with osteosarcopenia undergoing coronary revascularization through bypass surgery, an incidence of 28.6% of postoperative complications was observed, including infections, hemorrhages requiring resternotomy, pneumothorax, and hydrothorax, compared to 15.4% in patients without osteosarcopenia (Bazdyrev et al., 2022) (Fig. 4). Also, patients with osteosarcopenia undergoing emergency surgery for gastrointestinal perforation had a high percentage of events classified as Clavien-Dindo grade III or higher, with in-hospital mortality 33.3% *vs* 6.7% in those without osteosarcopenia (Fukushima et al., 2024) (Fig. 5).

### Criteria used to diagnose osteosarcopenia

**Figure 6:**
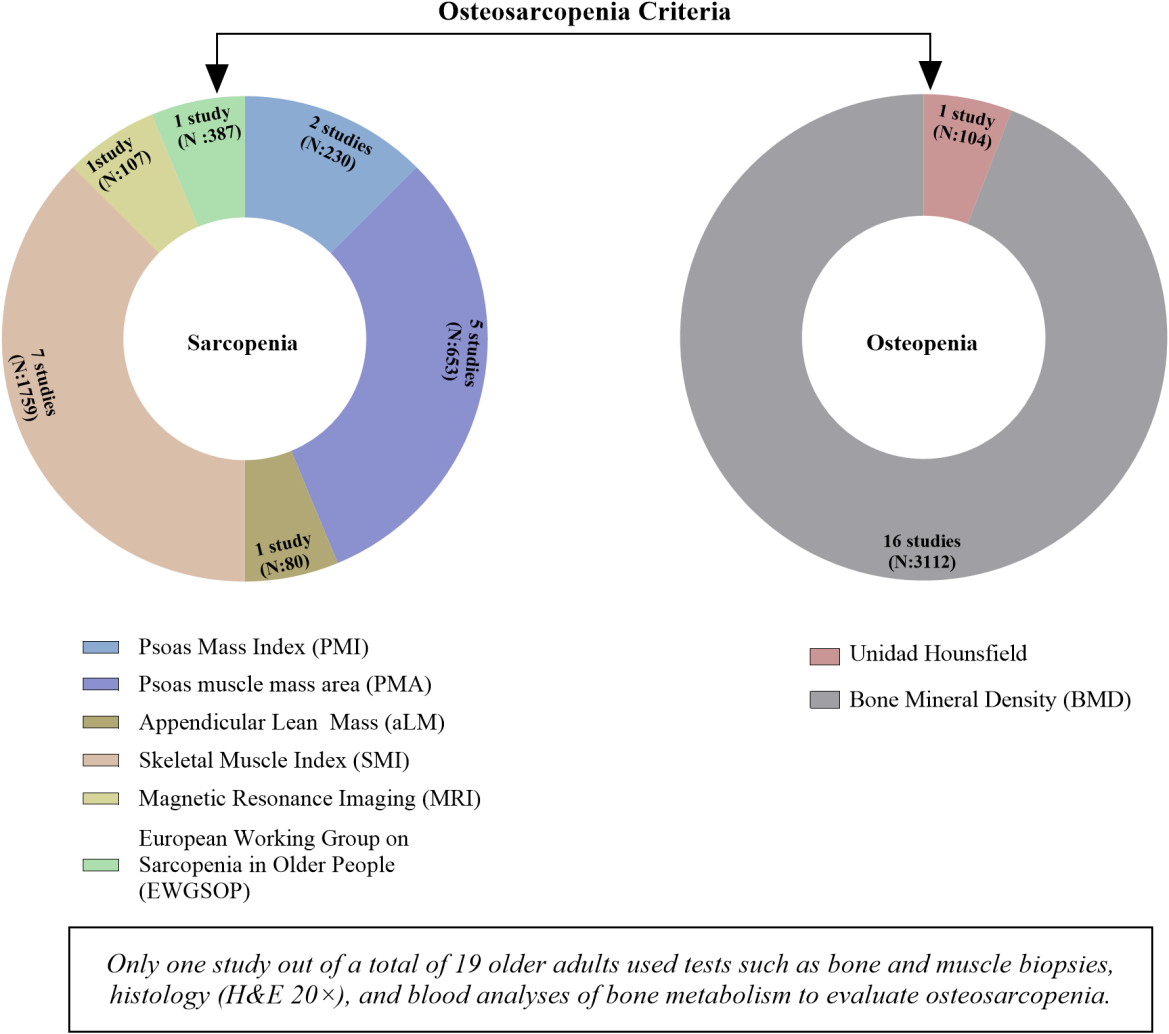
Criteria used for the diagnosis of osteosarcopenia. Note: The figure summarizes the criteria used to diagnose osteosarcopenia. The left ring (sarcopenia) indicates the studies and participants for which various assessment criteria were used (PMI, PMA, aLM, SMI, MRI, EWGSOP). Additionally, the right ring (osteopenia) shows the number of studies and sample sizes according to the measures used (Hounsfield Units and BMD).

Multiple methods were employed to assess osteosarcopenia across the included studies (Abe et al., 2024; Abe et al., 2023; Bae & Moon, 2020; Bazdyrev et al., 2021; Di Monaco et al., 2022; Fujimoto et al., 2024; Fukushima et al., 2024; Furukawa et al., 2021; Furukawa et al., 2023; Haffer et al., 2023; Kara & Ozturk, 2023; Matsumoto et al., 2024; Takano et al., 2023; Taniai et al., 2023; Yanagaki et al., 2023; Yanagaki et al., 2024). Sarcopenia was diagnosed using several approaches, the most common being the Skeletal Muscle Mass Index (SMI), which was used in seven studies with a total of 1,759 patients (Abe et al., 2024; Abe et al., 2023; Fujimoto et al., 2024; Takano et al., 2023; Yanagaki et al., 2023; Yanagaki et al., 2024). SMI was consistently measured at the level of the third lumbar vertebra (Abe et al., 2024; Abe et al., 2023; Takano et al., 2023; Yanagaki et al., 2023; Yanagaki et al., 2024), using cutoff values of <30 cm^2^/m^2^ for women and <40 cm^2^/m^2^ for men (Fujimoto et al., 2024), along with other criteria of <38 cm^2^/m^2^ for women and <42 cm^2^/m^2^ for *men* (Abe et al., 2024). The Psoas Muscle Area (PMA) was used in five studies with a total of 653 participants (Fukushima et al., 2024; Furukawa et al., 2021; Furukawa et al., 2023; Matsumoto et al., 2024; Taniai et al., 2023), also measured at the third lumbar vertebra (Fukushima et al., 2024; Furukawa et al., 2021; Furukawa et al., 2023; Matsumoto et al., 2024; Taniai et al., 2023). In two studies with a total of 230 participants, sarcopenia was assessed using the Psoas Muscle Index (PMI) (Bae & Moon, 2020; Kara & Ozturk, 2023), measured at the third lumbar vertebra (Kara & Ozturk, 2023); Other less commonly used methods included appendicular lean mass (ALM), used in one study with 80 patients (Di Monaco et al., 2022), and functional cross-sectional area (fCSA), which included a total of 387 patients (Haffer et al., 2023) (Fig. 6).

Regarding the measurement of osteopenia, 16 studies involving a total of 3,216 older adults assessed bone mineral density (BMD) (Abe et al., 2024; Abe et al., 2023; Bae & Moon, 2020; Bazdyrev et al., 2022; Di Monaco et al., 2022; Fujimoto et al., 2024; Fukushima et al., 2024; Furukawa et al., 2021; Furukawa et al., 2023; Haffer et al., 2023; Hirase et al., 2025; Matsumoto et al., 2024; Takano et al., 2023; Taniai et al., 2023; Yanagaki et al., 2023; Yanagaki et al., 2024), which was measured at the thoracic vertebra 11 (Th11) (Abe et al., 2023; Fujimoto et al., 2024; Fukushima et al., 2024; Furukawa et al., 2021; Matsumoto et al., 2024; Taniai et al., 2023; Yanagaki et al., 2023; Yanagaki et al., 2024); additionally, in a single study involving 104 older adults, the Hounsfield Unit (HU) was used as the measurement unit (Kara & Ozturk, 2023) (Fig. 6).

Lastly, only one study employed additional tests for the detection of osteosarcopenia through further analyses, such as bone and muscle biopsies (particularly of the biceps and quadriceps), specialized histological analyses (hematoxylin-eosin staining at 20x magnification), magnetic resonance imaging assessments, and blood tests for bone metabolism (calcium, phosphorus, vitamin D) (Bottai et al., 2023). Although diagnostic methodologies may vary among studies, computed tomography stands out as the most consistent and widely used method for evaluating both muscular and skeletal components in the diagnosis of osteosarcopenia (Fig. 6).

## Discussion

This scoping review mapped the existing literature on the prevalence, diagnostic criteria, and clinical impact of osteosarcopenia on postoperative outcomes in older adults undergoing major surgery. In addition, the review also categorized the evidence base according to study design, sample characteristics, geographic origin, and diagnostic methods. The findings indicate that the prevalence of osteosarcopenia varies widely across populations and surgical contexts, while substantial heterogeneity exists in the diagnostic approaches employed. Importantly, the evidence consistently shows that osteosarcopenia is associated with adverse postoperative outcomes, including increased complications, prolonged hospital stays, greater dependency, and higher mortality.

Osteosarcopenia is typically characterized by a relationship between the skeletal system and muscle tissue (Kara & Ozturk, 2023), in which variables such as physical inactivity and malnutrition contribute to the deterioration of both tissues, thereby impairing the body’s ability to recover following surgery (Clynes et al., 2021; Hirschfeld, Kinsella & Duque, 2017; Kara & Ozturk, 2023). From a pathophysiological perspective, chronic low-grade inflammation (“inflammaging”), hormonal dysregulation (including estrogen, testosterone, and growth hormone decline), and impaired bone–muscle crosstalk mediated by myokines and osteokines accelerate the simultaneous loss of bone density and muscle mass (Nusrat et al., 2025). Evidence from recent studies has shown that, in older adults with chronic conditions such as chronic kidney disease or chronic obstructive pulmonary disease, these mechanisms have been linked to the presence of osteosarcopenia and to the worse prognosis associated with this geriatric syndrome (Caldiroli et al., 2025; Lippi et al., 2022). In surgical contexts, these mechanisms are exacerbated by perioperative immobility, catabolic stress, and reduced nutritional intake, ultimately leading to higher risks of infection, delayed wound healing, and increased mortality (Abe et al., 2023; Matsumoto et al., 2024).

In the present scoping review, it was determined that the prevalence of osteosarcopenia varies according to patient characteristics such as age and geographic location, with higher prevalence observed among older Asian adults (Abe et al., 2024; Abe et al., 2023; Bae & Moon, 2020; Fujimoto et al., 2024; Fukushima et al., 2024; Furukawa et al., 2021; Furukawa et al., 2023; Matsumoto et al., 2024; Takano et al., 2023; Taniai et al., 2023; Yanagaki et al., 2023; Yanagaki et al., 2024). Previous studies have indicated that factors such as sex, geographic location, and lifestyle habits may significantly influence osteosarcopenia rates within the population (Chen et al., 2024; Huang et al., 2023). Similarly, in countries such as Japan, the older adult population has been increasing over recent decades (Arai et al., 2015), which has driven greater interest in the study of geriatric syndromes (Hosokawa et al., 2023), Therefore, it is essential to include demographic profiles and lifestyle characteristics of older adults to assess the risk of osteosarcopenia and to plan interventions aimed at reducing postoperative complications.

The review showed that the prevalence of osteosarcopenia may vary and increase the risk factor for postoperative recovery, depending on the type of surgical intervention performed (Matsumoto et al., 2024; Wang et al., 2024). In oncological surgeries, patients with osteosarcopenia consistently exhibited higher postoperative mortality, poorer nutritional status, more aggressive tumor progression, and reduced survival times compared with non-osteosarcopenic patients (Abe et al., 2024; Matsumoto et al., 2024; Taniai et al., 2023). Studies suggest that these complications may be attributed to elevated inflammation and lower protein levels associated with osteosarcopenia (Bottai et al., 2023), contributing to a higher risk of infections, impaired wound healing, and prolonged hospital stays (Abe et al., 2024; Abe et al., 2023; Darling, 2022; Huang, Li & Leng, 2022; Kositsawat, Duque & Kirk, 2021; Matsumoto et al., 2024; Musio et al., 2023; Polito et al., 2022). Added to this issue, oncology patients often face a nutrient absorption problem due to cancer treatment, which further weakens their immune system (Casabella et al., 2024; Coccolini et al., 2021; Meza-Valderrama et al., 2021). These findings highlight the importance of early interventions to improve patients’ physical and nutritional condition prior to oncological surgery, particularly in those with osteosarcopenia, to reduce complications and improve long-term outcomes (Abe et al., 2024; Matsumoto et al., 2024).

Previous reviews have shown that patients with osteosarcopenia present a higher risk of falls, fractures, and greater frailty in the older adult population (Polito et al., 2022; Teng et al., 2021), this mainly due to the decrease in static balance (Debruin et al., 2024; Lee et al., 2024) and physical function, causing a musculoskeletal compromise, along with a more challenging rehabilitation process in older adults post-operated for orthopedic surgeries (Debruin et al., 2024; Hurtado et al., 2024; Inoue et al., 2022; Lee et al., 2024; Rosas-Carrasco et al., 2024). Likewise, the studies included in this review showed that osteosarcopenia amplifies the risk of infections, delays wound healing, and increases postoperative complications in older adults after orthopedic surgeries (Bae & Moon, 2020; Bottai et al., 2023; Di Monaco et al., 2022; Furukawa et al., 2023; Kara & Ozturk, 2023). Specifically, hip fracture patients with osteosarcopenia demonstrated slower recovery, greater disability, higher risk of new fractures, and increased mortality, while those undergoing vertebral fracture surgery exhibited greater bone fragility, fat infiltration of paraspinal muscles, and higher in-hospital mortality (Bae & Moon, 2020; Di Monaco et al., 2022; Haffer et al., 2023; Kara & Ozturk, 2023). These findings underscore the importance of preoperative screening for osteosarcopenia in patients undergoing orthopedic procedures to reduce complications and improve recovery trajectories.

In gastrointestinal and cardiovascular surgeries, osteosarcopenia has been associated with different types of complications like bleeding and higher mortality rates (Bazdyrev et al., 2022; Fukushima et al., 2024). In addition, the literature suggests that there is also a negative impact on metabolic stress and the patient’s nutritional status following gastrointestinal surgeries (Carey et al., 2011; Fukushima et al., 2024; Narendra et al., 2020), these two factors are essential for protein synthesis required for the repair of damaged tissues (Barchitta et al., 2019). Likewise, patients recovering from cardiac interventions face a higher risk of cardiac complications, conditions that are linked to a lack of mobilization (Bazdyrev et al., 2022; Lund et al., 2016). Specifically, in gastrointestinal emergencies such as perforations, patients with osteosarcopenia experienced more severe postoperative complications and markedly higher in-hospital mortality, while in cardiac revascularization surgery, osteosarcopenia was linked to higher rates of infections, hemorrhage, and other serious complications compared with non-osteosarcopenic patients (Fukushima et al., 2024). These interrelationships suggest that osteosarcopenia predisposes patients to a wide range of metabolic, systemic, infectious, respiratory, and other complication (Chen et al., 2024; Park et al., 2024). Additionally, aging promotes the onset of chronic diseases such as diabetes, cardiovascular disorders, inflammatory conditions, and others, which not only affect the patient’s overall health but also contribute to the general risk of musculoskeletal diseases (Murray & Coleman, 2019; Thorpe et al., 2021; Valderrábano & Wu, 2019). Additional risk factors, including low physical activity, poor nutritional status, and anemia, further accelerate the progression of bone and muscle loss, ultimately leading to osteosarcopenia (Oliveira et al., 2022; Valderrábano & Wu, 2019). While previous studies have proposed that multimodal interventions combining exercise and nutritional support may mitigate the progression of osteosarcopenia (Kirk, Zanker & Duque, 2020; Lichtenberg et al., 2019), no studies have yet evaluated such approaches in surgical populations. Therefore, our findings emphasize the need for future research to investigate the feasibility and effectiveness of these strategies in patients undergoing major surgery.

On the other hand, the findings of this review highlight that the prevalence of osteosarcopenia varies considerably depending on diagnostic methods. While established criteria exist that link osteoporosis (assessed *via* bone mineral density) and sarcopenia (measured through skeletal muscle index), a unified diagnostic protocol for osteosarcopenia remains undeveloped (Cedeno-Veloz, López-Dóriga Bonnardeauxa & Duque, 2019; Yoo & Ha, 2018). Furthermore, imaging methods like dual-energy X-ray absorptiometry offer high diagnostic accuracy (Ariza Galindo et al., 2022; Kirk, Zanker & Duque, 2020), however, their high costs and limited accessibility present barriers to routine implementation in public health settings. Therefore, establishing cost-effective and widely applicable diagnostic protocols is essential to improving osteosarcopenia management in diverse clinical settings.

Several significant limitations and gaps emerge that future studies should address. First, the study focused on the impact of osteosarcopenia on surgical complications, overlooking a comparison with different populations. Second, the current literature was biased toward Asian populations, which likely reflects the demographic and research context of these countries, where rapidly aging populations, higher prevalence of osteosarcopenia, and stronger investment in geriatric and oncological research have resulted in a greater volume of scientific output compared with other regions. Third, several methods are used to diagnose osteosarcopenia, but there is no universal cutoff point or single standard, which complicates comparisons between studies. The establishment of uniform diagnostic criteria for osteosarcopenia would improve the comparability and clinical utility of findings. Fourth, most studies in this field were cross-sectional, which limits the ability to establish causality for osteosarcopenia. Future longitudinal studies would allow researchers to explore causal relationships between osteosarcopenia and clinical outcomes in this population. Finally, there is a concentration of studies on osteosarcopenia in certain cancer types (such as hepatocellular carcinoma and colorectal cancer) and surgical settings (hip and vertebral fractures), which may be explained by their high clinical burden, public health relevance, and associated mortality and disability, making them priority areas for surgical and geriatric research. In contrast, other cancers and surgical contexts remain underexplored, likely due to lower perceived clinical priority, limited recognition of osteosarcopenia, or restricted research resources.

## Conclusions

In this review, it was concluded that the prevalence of osteosarcopenia varies depending on population characteristics such as age, geographic location, and the type of surgical intervention performed. This syndrome has also been associated with a higher incidence of postoperative complications. In oncological surgeries, osteosarcopenia was associated with increased postoperative mortality, reduced survival, and poorer nutritional status. In orthopedic surgeries, it was linked to delayed recovery, higher rates of new fractures, functional decline, and increased mortality. In cardiovascular and gastrointestinal surgeries, osteosarcopenia was associated with higher rates of severe complications, including infections, bleeding, delayed wound healing, and greater in-hospital mortality. Furthermore, variations in diagnostic criteria are highlighted, with the most common approach being the coexistence of sarcopenia and osteopenia. Despite these findings, important gaps persist: most evidence is derived from Asian populations, with limited representation from Europe, North America, and Latin America; studies are concentrated on certain cancers and trauma-related surgeries, leaving other surgical contexts underexplored; and the predominance of retrospective designs limits causal inference. These gaps emphasize the urgent need for standardized diagnostic protocols and future longitudinal and interventional research that includes more diverse populations and surgical scenarios.

## Supplemental Information

10.7717/peerj.20527/supp-1 Supplemental Information 1 Preferred Reporting Items for Systematic reviews and Meta-Analyses extension for Scoping Reviews (PRISMA-ScR) Checklist

10.7717/peerj.20527/supp-2 Supplemental Information 2 Search Strategy

## Additional Information and Declarations

### Data Availability

The following information was supplied regarding data availability: Raw data were not generated in this literature review.
