# Supplementary material for: Impact of osteosarcopenia in older people on prognosis following major surgery: a scoping review
Source: PeerJ. 2026 Jan 8;14:e20527. doi: 10.7717/peerj.20527 (PMC12790780; doi:10.7717/peerj.20527)
Supplement: Supplemental Information 2 [file peerj-14-20527-s002.docx]

**Supplementary Table S1: Search Strategy**

| Database  Search: August 18, 2025 | Total |
| --- | --- |
| MEDLINE (Pubmed) |  |
| ((osteosarcopenia[Title/Abstract]) OR (osteosarcopenic*[Title/Abstract])) AND ((((((((((general surgery[MeSH Terms]) OR (Thoracic Surgery[MeSH Terms])) OR (Acute Care Surgery[MeSH Terms])) OR (Orthopedic Procedures[MeSH Terms])) OR (Minor Surgical Procedures[MeSH Terms])) OR (Enhanced Recovery After Surgery[MeSH Terms])) OR (Hospitalization[MeSH Terms])) OR (surgery[Title/Abstract])) | 34  Documents |
| SCOPUS |  |
| ( ( TITLE-ABS-KEY ( surgery AND general ) OR TITLE-ABS-KEY ( surgery ) OR TITLE-ABS-KEY ( surgery AND Procedures ) OR TITLE-ABS-KEY ( surgery AND  Recovery ) OR TITLE-ABS-KEY ( Orthopedic AND procedures ) ) ) AND ( ( TITLE-ABS-KEY ( osteosarcopenia ) OR TITLE-ABS-KEY ( osteosarcopenic ) | 45 Documents |
| Web of Science |  |
| (ALL=(general surgery (All Fields) or thoracic Surgery (All Fields) or Orthopedic Procedures (All Fields) or Enhanced Recovery After Surgery (All Fields) or Minor Surgical Procedures (All Fields) or Hospitalization (All Fields))) AND ALL=(osteosarcopenia) | 68 Documents |
| Scielo |  |
| ((osteosarcopenia) OR (osteosarcopenic))  AND  (Surgery) | 5 Documents |
| PEDRO  ((osteosarcopenia) OR (osteosarcopenic)) | 12  Documents |
